# Beraprost and Overall Survival in Cats with Chronic Kidney Disease

**DOI:** 10.3390/vetsci10070459

**Published:** 2023-07-13

**Authors:** Hiroyuki Ito, Takumi Matsuura, Tadashi Sano

**Affiliations:** 1 Ichikawa General Hospital, Kariya Animal Hospital Group, Chiba 272-0034, Japan; 2 Toray Industries, Inc., Tokyo 103-8666, Japan; 3 School of Veterinary Medicine, Rakuno Gakuen University, Hokkaido 069-8501, Japan

**Keywords:** beraprost, feline, chronic kidney disease, progression-free survival, overall survival

## Abstract

**Simple Summary:**

Chronic kidney disease (CKD) is a highly prevalent disorder in senior cats. CKD is commonly diagnosed together with other disorders; therefore, overall survival, based on all-cause death, including complications, is the most important outcome for treatment response in feline CKD. Although researchers have been seeking a pharmaceutical to improve overall survival, unfortunately, they have not discovered it yet. Beraprost (also called beraprost sodium or BPS) is a prostacyclin analogue that has a vasoprotective effect, which is unique and completely different from renin–angiotensin inhibitors. This study demonstrated that beraprost therapy was associated with better overall survival, and the findings shed light on cats suffering with CKD, their owners, and veterinarians in clinical practice.

**Abstract:**

Background: Overall survival is the most important outcome for treatment response in feline chronic kidney disease (CKD). Beraprost has been shown to reduce the kidney function decline in cats with International Renal Interest Society (IRIS) stage 2 and 3 CKD. However, the association with prolonged survival has not yet been examined. Objective: To assess the relationship between beraprost and overall survival in cats with CKD in real clinical practice. Animals: Client-owned cats with IRIS stage 3 CKD (*n* = 134) were evaluated between 2017 and 2020. Methods: A retrospective cohort study based on data from electronic medical records of one hospital. Results: The cohort was divided into “beraprost therapy” and “no beraprost therapy” groups, and survival analyses revealed that overall survival was significantly longer in the beraprost therapy group, using Kaplan–Meier curves (*p* = 0.004). However, baseline phosphate is known to be an important prognostic indicator and was not well balanced between the two groups. Therefore, a subcohort of 97 cats was selected (those having baseline phosphate <6.0 mg/dL) that allowed for this parameter to be balanced between groups. The survival data in this subcohort were consistent with those of the overall study cohort. Conclusions: In feline patients with CKD, beraprost therapy is associated with better overall survival.

## 1. Introduction

Chronic kidney disease (CKD) is a common disease worldwide and a leading cause of mortality and morbidity in older cats. Advanced disease is characterized by incurable kidney function decline and poor prognosis [1,2]. Cardiac function can be impaired by the presence of decreased kidney function in cats, which is known as cardiovascular–renal disorder (CvRD) [3]; a subset of cats with CKD can die from heart failure. In addition, the complications of feline CKD include hypertension, proteinuria, hypokalemia, hyperphosphatemia, urinary tract infections, anemia, and CKD-related mineral bone disorders [1,2]. Hyperthyroidism [4] and diabetes mellitus [5] also occur as co-morbidities. All such disorders can also contribute to early mortality. Therefore, overall survival, based on all-cause death, including complications and co-morbidities, is the most important outcome for treatment response.

Vascular endothelial function is systemically impaired from an early stage of CKD. At the same time, endothelial damage causes a progressive loss of kidney microvasculature in the tubulointerstitium, which leads to local hypoxia, induction of pre-fibrotic responses, scarring, and deterioration of kidney function [6,7]. Although there is no direct evidence in cats with CKD, altered endothelial function in cats with CKD can be predicted given increased plasma asymmetric dimethylarginine, an endogenous inhibitor of nitric oxide (NO) production [8]. Beraprost is a prostacyclin analogue that has a protective effect on injured endothelial cells [9]. Beraprost induces endothelial NO synthase (eNOS) and increases NO production in vascular endothelial cells (mouse, human, and bovine) to improve endothelial function [10] and enhances hepatocyte growth factor expression to provide endothelial cytoprotective effects in a diabetic rat model [11]. In preclinical models of CKD, beraprost has been shown to inhibit reduction in kidney function and improve survival in nephritic [12] and partially nephrectomized rats [13].

In a randomized, double-blind, placebo-controlled trial, beraprost was reported to suppress the deterioration of kidney function, as measured by increased serum creatinine in cats with International Renal Interest Society (IRIS) stages 2 and 3 CKD. Beraprost also showed positive effects on appetite, dehydration, owner-reported quality of life (QOL) evaluations, and overall veterinary evaluations of a treatment’s effects; however, in that trial, efficacy for delaying disease progression or prolonging survival was not evaluated, standard therapy was partly restricted, and the cats with coexisting disorders were excluded [14].

The purpose of this retrospective cohort study was to describe the relationship between beraprost and overall survival in cats with CKD in real clinical practice.

## 2. Materials and Methods

### 2.1. Case Selection

Medical records of the Ichikawa General Hospital, Kariya Animal Hospital Group were searched to identify all cats that had blood and urine collected for serum creatinine analysis (*n* = 1681) and urinalysis (*n* = 1399) between April 2017 and December 2020. In this hospital, all test results, death records, and medication records had been archived with the electronic medical record system Ahmics (PetCommunications Co., Ltd., Osaka, Japan) since before 2017. Cats were included in the study if their medical records confirmed a diagnosis of CKD, based on: elevated serum creatinine, determined on two separate occasions not less than 7 days apart; urine specific gravity <1.035, with or without persistent proteinuria and other clinical pathology parameters; compatible history (e.g., polyuria polydipsia); and physical examination and/or diagnostic imaging findings (e.g., palpably small kidneys). Staging was undertaken according to the IRIS staging of CKD (modified 2019) [2]. Data were collected on baseline characteristics: age, bodyweight, sex, neuter status, breed, and therapies being used to manage the CKD. The distribution of IRIS stage and beraprost prescription in the CKD-diagnosed cats was shown in Table 1. A pilot analysis of all CKD cats was performed on these data and the baseline clinical pathology results to determine homogeneity of baseline characteristics between those treated with beraprost and those not within each IRIS stage 1–4. IRIS stage 3 (*n* = 134, defined as cohort A) cats were well-balanced for most baseline characteristics, and, therefore, only these cats were subjected to further analysis. Additionally, a subcohort of cohort A with baseline phosphate <6.0 mg/dL was defined as cohort B.

### 2.2. Study Design

The start date for all time-to-event outcomes was the date of first documented prescription of beraprost (RAPROS^®^, Toray Industries, Inc., Tokyo, Japan) in the beraprost therapy group or the staging results in the no beraprost therapy group. Data on baseline characteristics and coexisting disorders were extracted on or before the start date. Progression of disease was defined in this study as a ≥25% increase in serum creatinine, compared to baseline, based on previously published data [15]. Progression-free survival was calculated and defined as the time from the start date until the date of progression, death from any cause, euthanasia, or last follow-up. Overall survival was measured from the start date until the date of death from any cause, euthanasia, or last follow-up. Discontinuation was defined as lost follow-up due to no further medical records. The incidence of chronic disorders developing during the study was also assessed.

### 2.3. Data Analysis

Baseline characteristics, coexisting disorders, and the incidence of chronic disorders were summarized and compared between the groups. Quantitative variables were characterized by their means, medians, 25th and 75th percentile (Q1 and Q3), and standard deviations, whereas the qualitative variables were according to the percentages of cats. The differences between the groups were tested by Mann–Whitney U test or Welch’s t-test for quantitative variables and Fisher’s exact test for qualitative variables. With regard to main-effect covariates: for beraprost and baseline characteristics (urea, phosphate, calcium, and urine protein), multivariable Cox regression models were constructed using forward stepwise selection. Only covariates with a multivariate *p* < 0.10 for beraprost and baseline characteristics by Wald test were retained. The veracity of the proportional-hazards assumption in the models was assessed by inspecting log–log plots of adjusted survival curves for parallel lines, and no violation of the assumptions was observed. Progression-free survival and overall survival were assessed with the Kaplan–Meier method and compared with a log-rank test. Hazard ratio for disease progression or death, summary of events (disease progression, treatment discontinuation, death, euthanasia), progression-free survival at 1 year with SE and 95% CI, overall survival at 1 and 3 years with SE and 95% CI, and median progression-free and overall survival with SE and 95% CI were analyzed. In order to demonstrate consistency of the treatment effect in subgroups, progression-free and overall survival analyses were performed, in which the hazard ratio and 95% CI were calculated using an unstratified Cox model, and *p* values were determined by Wald test. A commercial statistical software program was used for all analyses (BellCurve for Excel; Social Survey Research Information Co., Ltd., Tokyo, Japan). Statistical significance was set at 2-sided * *p* < 0.05, ** *p* < 0.01.

## 3. Results

### 3.1. Baseline Characteristics of All Cats in Cohort A

One hundred and thirty-four cats with IRIS stage 3 CKD were enrolled in the study (cohort A). Baseline characteristics are presented in Table 2. The results (means, medians, numbers, and percents) demonstrated geriatric age (median, 15.3 years), frequently neutered (115 cats, 85.8%), mainly domestic shorthair breed (91 cats, 67.9%), increased creatinine (median, 3.3 mg/dL), urea (median, 42.0 mg/dL), decreased urine specific gravity (median, 1.014) at the time or baseline assessment, mostly receiving subcutaneous fluid therapy (106 cats, 79.1%), and agents for managing inappetence, nausea, and vomiting (102 cats, 76.1%) during the study. At the time of the start date, coexisting disorders were present in 38 cats (28.4%), the most observed was hyperthyroidism (13 cats, 9.7%), and the second was congestive heart failure (10 cats, 7.5%). The number of cats prescribed beraprost was 57 cats (42.5%), and the mean dose of beraprost was 15.0 µg/kg twice daily (Table 2).

To address the effects of beraprost on survival in further depth, cohort A (*n* = 134) was divided into two groups, a beraprost therapy group (*n* = 57) and a no beraprost therapy group (*n* = 77), and a retrospective analysis was carried out. The groups were matched for the distribution over time; the median start date was 3 July 2018 in the beraprost therapy group and 30 June 2018 in the no beraprost therapy group. Baseline characteristics are presented in Table 2. The results revealed that urea, phosphate, and urine protein were significantly higher in the no beraprost therapy (*p* < 0.01 for urea, and phosphate, *p* < 0.05 for urine protein by Mann–Whitney U test), and calcium was significantly higher in the beraprost therapy group (*p* < 0.05 by Mann–Whitney U test). In the previous study on the prognostic factors in cats with chronic kidney disease, when the subgroups with phosphate > 4.7 to ≤6.8 and ≤4.7 mg/dL were compared, higher phosphate was shown to be significantly (*p* < 0.001) associated with shorter kidney survival time [16]. Thus, it should be noted in the present study that the difference in baseline phosphate between the two groups could have influenced the survival analysis results of cohort A.

### 3.2. Multivariable Analyses of All Cats in Cohort A

Among the 134 cats with IRIS CKD stage 3 in cohort A, the mean duration of follow-up for progression-free survival was 10.9 months (95% CI, 9.0 to 12.8), disease progression occurred in 86 cats (64.2%), and discontinuation in 48 (35.8%). During the mean follow-up of 12.8 months (95% CI, 10.8 to 14.8) for overall survival, all-cause death occurred in 80 cats (59.7%), euthanasia in 3 (2.2%), and discontinuation in 61 (45.5%).

In order to exclude confounding factors that could influence the treatment effect of beraprost, a multivariable model was constructed from the baseline variables (urea, phosphate, calcium, and urine protein) with beraprost as covariates. Two factors were identified that correlated with disease progression; HR of beraprost was 0.59 (*p* = 0.032), and urea was 2.90 (*p* < 0.001). Likewise, three factors for overall survival were found; HR of beraprost was 0.58 (*p* = 0.047), urea was 2.61 (*p* = 0.004), and phosphate was 2.41 (*p* = 0.056). HRs of the factors other than beraprost were >1.00, associated with poorer prognosis. Collectively, these results demonstrated that beraprost was the only study treatment correlated significantly with improvement in progression-free and overall survival, respectively (Table 3).

### 3.3. Survival Analyses of the Two Groups in Cohort A

**Figure 1:**
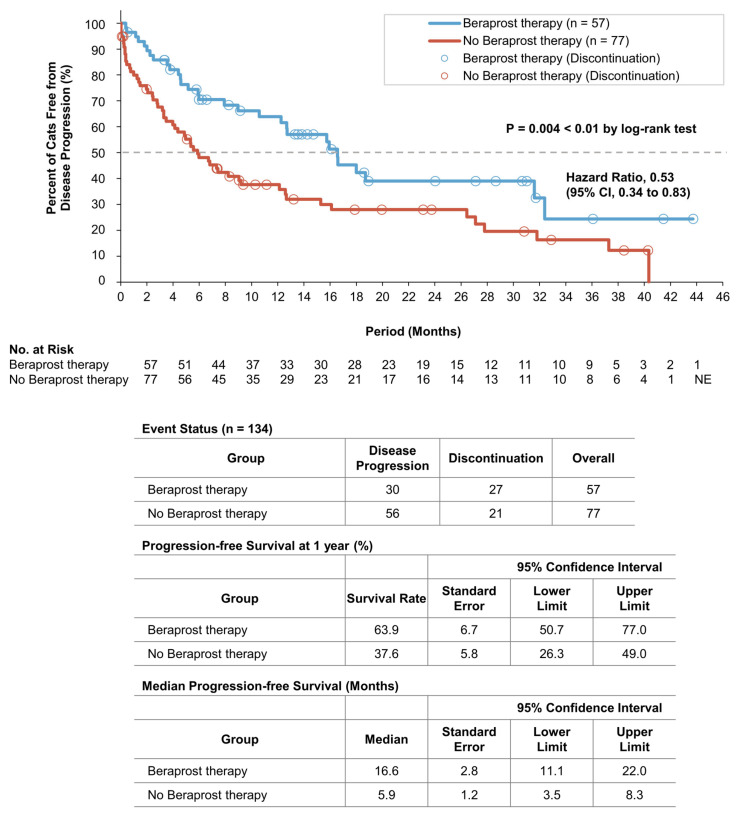
Progression-free survival in cohort A: IRIS CKD stage 3 (creatinine: 2.9–5.0 mg/dL). Kaplan–Meier estimates of progression free-survival, summary of events, progression-free survival at 1 year, and median progression-free survival. *p* value was calculated by two-sided log-rank test. Disease progression events in the beraprost therapy group included death (in 13 cats); disease progression events in the no beraprost therapy group included death (in 19 cats) and euthanasia (in 1 cat). CI denotes confidence interval, and NE could not be evaluated.

At the time of the final analysis of progression-free survival, the mean durations of follow-up were 13.2 months for the beraprost therapy group and 9.3 for the no beraprost therapy group. A total of 30 cats (52.6%) in the beraprost therapy group and 56 (72.7%) in the no beraprost therapy group progressed. Progression-free survival was significantly longer with beraprost therapy (*p* = 0.004 by log-rank test); the estimated rates of progression-free survival at 1 year were 63.9% (SE, 6.7, 95% CI, 50.7 to 77.0) in the beraprost therapy group and 37.6% (SE, 5.8, 95% CI, 26.3 to 49.0) in the no beraprost therapy group. The median progression-free survival was 16.6 months (SE, 2.8, 95% CI, 11.1 to 22.0) in the beraprost therapy group and 5.9 months (SE, 1.2, 95% CI, 3.5 to 8.3) in the no beraprost therapy group (Figure 1).

**Figure 2:**
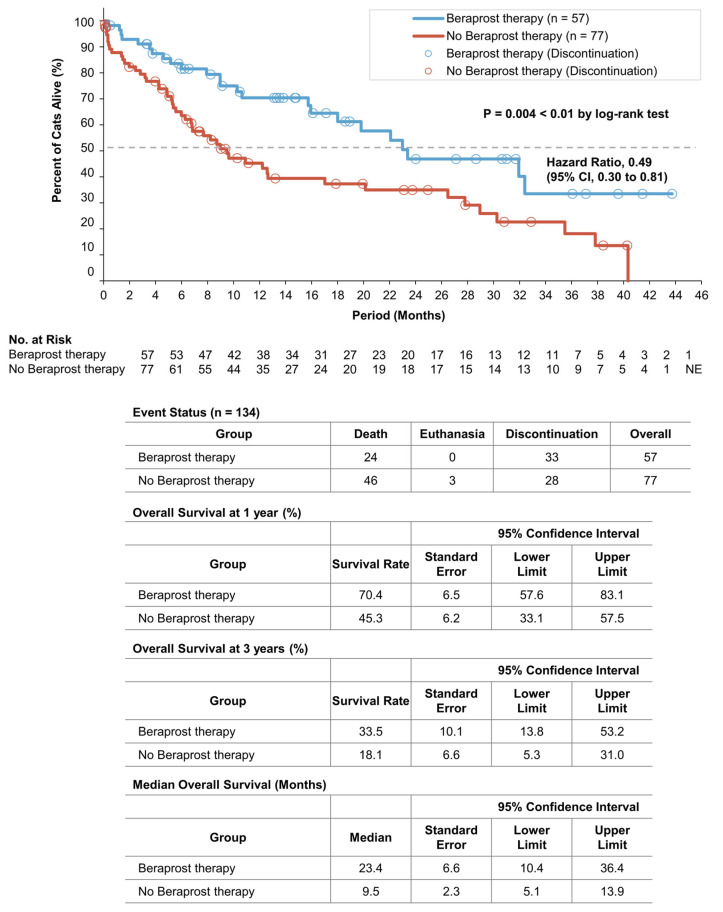
Overall survival in cohort A: IRIS CKD stage 3 (creatinine: 2.9–5.0 mg/dL). Kaplan–Meier estimates of overall survival, summary of events, overall survival at 1 and 3 years, and median progression-free survival. *p* value was calculated by two-sided log-rank test. CI denotes confidence interval, and NE could not be evaluated.

A total of 24 cats (42.1%) in the beraprost therapy group and 49 (63.6%) in the no beraprost therapy group died, which included 3 cats euthanized in the no beraprost therapy group. Overall survival was significantly longer with beraprost therapy; the estimated rates of overall survival at 1 year were 70.4% (SE, 6.5, 95% CI, 57.6 to 83.1) in the beraprost therapy group and 45.3% (SE, 6.2, 95% CI, 33.1 to 57.5) in the no beraprost therapy group; at 3 years, they were 33.5% (SE, 10.1, 95% CI, 13.8 to 53.2) in the beraprost therapy group and 18.1% (SE, 6.6, 95% CI, 5.3 to 31.0) in the no beraprost therapy group (*p* = 0.004 by log-rank test). The median overall survival was 23.4 months (SE, 6.6, 95% CI, 10.4 to 36.4) in the beraprost therapy group and 9.5 months (SE, 2.3, 95% CI, 5.1 to 13.9) in the no beraprost therapy group (Figure 2).

Furthermore, to examine the robustness of the findings, cohort A was divided into various subgroups based on baseline variables, and unstratified Cox regression analyses were carried out for each subgroup. All the HRs of the beraprost therapy group compared to the no beraprost therapy group were significantly better for progression free and overall survival (*p* = 0.003 to 0.042), and there were no interactions between the treatment effect of beraprost and any of the subgroup variables (Table 4).

### 3.4. Baseline Characteristics of All Cats in Cohort B

There remained a concern with the retrospective analysis of cohort A that baseline phosphate was significantly higher in the no beraprost therapy group, and this is known to be associated with poor prognosis of feline CKD. In order to solve the above concern, cats with phosphate ≥ 6.0 mg/dL were removed from cohort A, and another retrospective analysis was conducted. Ninety-seven (97) cats with IRIS stage 3 CKD and phosphate < 6.0 mg/dL were enrolled into cohort B, which was divided into the two groups: the beraprost therapy group (*n* = 47) and the no beraprost therapy group (*n* = 50). Baseline characteristics are presented in Table 5. Phosphate was well balanced between the two groups; the median was 4.3 mg/dL in the beraprost therapy group versus to 4.4 mg/dL in the no beraprost therapy group, but urea was significantly higher in the no beraprost therapy group (*p* < 0.05, Mann–Whitney U test), and calcium and kidney diet-other (other than Renal support and k/d) were significantly higher in the beraprost therapy group (*p* < 0.05, Welch T test, *p* < 0.05, Fisher’s exact test, respectively).

### 3.5. Survival Analyses of the Two Groups in Cohort B

**Figure 3:**
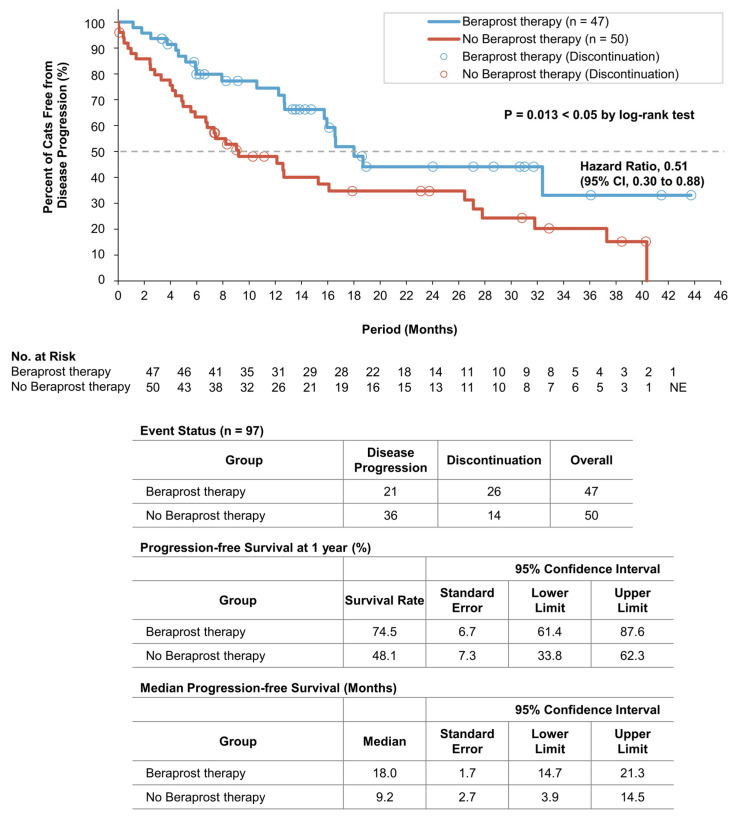
Progression-free survival in cohort B: IRIS CKD stage 3 (creatinine: 2.9–5.0 mg/dL) and phosphate <6.0 mg/dL. Kaplan–Meier estimates of progression free-survival, summary of events, progression-free survival at 1 year, and median progression-free survival. *p* value was calculated by two-sided log-rank test. Disease progression events in the beraprost therapy group included death (in 9 cats); disease progression events in the no beraprost therapy group included death (in 12 cats). CI denotes confidence interval, and NE could not be evaluated.

At the time of the final analysis of progression-free survival, the mean duration of follow-up was 13.6 months (14.8 months in the beraprost therapy group and 12.5 months in the no beraprost therapy group). A total of 21 cats (44.7%) in the beraprost therapy group and 36 (72.0%) in the no beraprost therapy group progressed. Progression-free survival was significantly longer in the beraprost therapy group (*p* = 0.013 by log-rank test); the estimated rates of progression-free survival at 1 year were 74.5% (SE, 6.7, 95% CI, 61.4 to 87.6) in the beraprost therapy group and 48.1% (SE, 7.3, 95% CI, 33.8 to 62.3) in the no beraprost therapy group. The median progression-free survival was 18.0 months (SE, 1.7, 95% CI, 14.7 to 21.3) in the beraprost therapy group and 9.2 months (SE, 2.7, 95% CI, 3.9 to 14.5) in the no beraprost therapy group (Figure 3).

**Figure 4:**
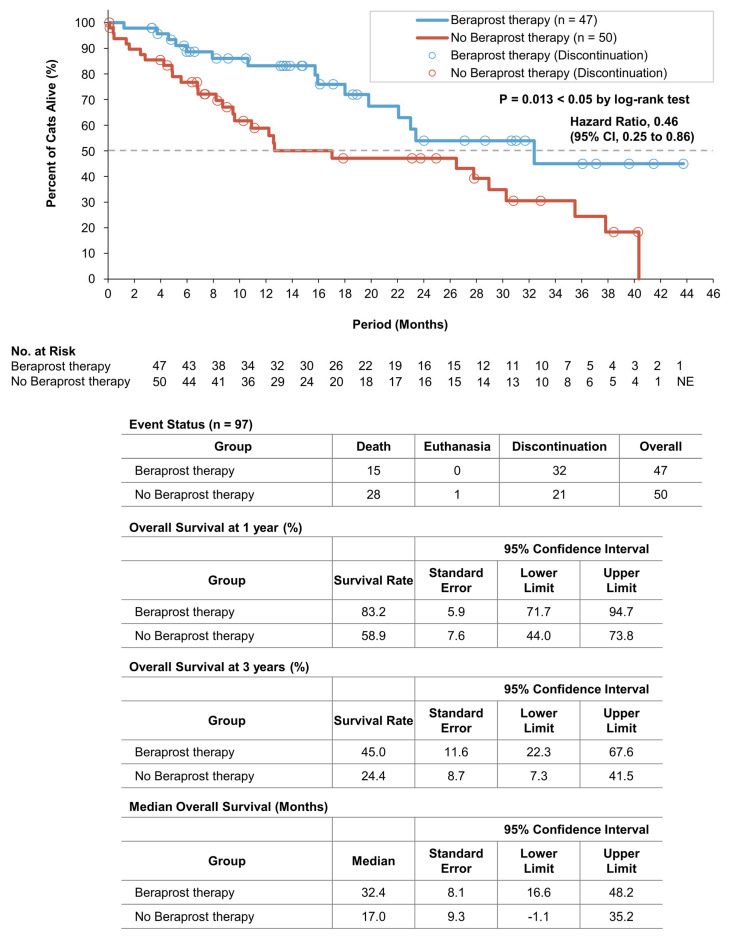
Overall survival in cohort B: IRIS CKD stage 3 (creatinine: 2.9–5.0 mg/dL) and phosphate < 6.0 mg/dL. Kaplan–Meier estimates of overall survival, summary of events, overall survival at 1 and 3 years, and median progression-free survival. *p* value was calculated by two-sided log-rank test. CI denotes confidence interval, and NE could not be evaluated.

A total of 15 cats (31.9%) in the beraprost therapy group and 29 (58.0%) in the no beraprost therapy group died, which included 1 cat euthanized in the no beraprost therapy group. Overall survival was significantly longer with beraprost therapy; the estimated rates of overall survival at 1 year were 83.2% (SE, 5.9, 95% CI, 71.7 to 94.7) in the beraprost therapy group and 58.9% (SE, 7.6, 95% CI, 44.0 to 73.8) in the no beraprost therapy group; at 3 years, these were 45.0% (SE, 11.6, 95% CI, 22.3 to 67.6) in the beraprost therapy group and 24.4% (SE, 8.7, 95% CI, 7.3 to 41.5) in the no beraprost therapy group (*p* = 0.013 by log-rank test). The median overall survival was 32.4 months (SE, 8.1, 95% CI, 16.6 to 48.2) in the beraprost therapy group and 17.0 months (SE, 9.3, 95% CI, −1.1 to 35.2) in the no beraprost therapy group (Figure 4). Taken together, these findings indicated that beraprost was an important factor associated with better survival in cohort B, in which any bias from difference in baseline phosphate had been excluded.

### 3.6. Onset of Chronic Disorders of the Two Groups in Cohort A

During the study, the onset of chronic disorders was found in 21 cats (15.7%) out of 134 cats: the most frequently observed were cardiovascular disorders (10 cats, 7.5%), and the second was neoplasia (6 cats, 4.5%); in other words, there was a higher incidence of cardiovascular–kidney complications than neoplasia. The overall incidence in the beraprost therapy group was 5 (8.8%), and it was 16 (20.8%) in the no beraprost therapy group (Table 6).

## 4. Discussion

Retrospective cohort studies have often been considered less valuable than randomized studies in scientifically demonstrating a causal relationship between factors and outcomes. The reasons are as follows: (A) Reporting bias, where outcomes are deliberately not reported or are partially reported; (B) Insufficiency of medical records, especially considering that the shortcomings of paper medical records have been recognized as limited in completeness and consistency; (C) Potential confounding variable bias, where non-randomized studies have difficulty avoiding confounding variables over a time period. However, non-randomized studies can complement randomized trials in long-term outcomes, rare events, and populations that are common in real-world practice [17,18]. In this study, multivariable Cox regression analysis for survival in 134 cats with IRIS stage 3 CKD (cohort A) showed beraprost only was related to better survival. Furthermore, the cohort was divided into a beraprost therapy group and a no beraprost therapy group, which were analyzed to further evaluate association between beraprost and survival in cats with CKD. This could complement previous information about the efficacy and safety of beraprost in cats with CKD that were demonstrated by a previous randomized study [14]. All data regarding test results, diagnoses, prescriptions, and death records were obtained from electronic medical records, so the potential biases were minimized. The evaluated outcomes were clear and the clinical implications straightforward. The robustness of the results was examined with Cox model sensitivity analyses in the various groups and further retrospective analyses.

The baseline profile in the overall study population was mostly consistent with those of previous studies: (A) a median age of 15.3 years versus the mean age 14.4 years of cats with uremic CKD [19], a mean age of 10.6 years of cats in IRIS stage 3 [16], and a median age of 13.0 years in cats with normotensive IRIS stage 3 CKD [20]; (B) a median weight of 3.6 kg versus a median weight of 3.8 kg in cats with IRIS stage 3 CKD [21], and a mean weight of 4.1 kg in cats in IRIS stage 3 [16]; (C) percentages of females and neutered were 46.3% and 85.8% versus the respective percentages of 38.5% and 97.4% in cats with uremic CKD [19], and these were 36.9% and 93.9% in cats in IRIS stage 3 [16]; (D) the most common breed was domestic shorthair, being 67.9%, versus the percentage of 74.4% of cats with uremic CKD [19] and 66.2% of cats in IRIS stage 3 [16]; (E) the mean creatinine was 3.4 mg/dL versus the mean 3.6 mg/dL in cats with uremic CKD [19] and 3.5 mg/dL [16]; (F) the mean packed cell volume was 34.5% versus the mean packed cell volume 30% in cats with uremic CKD [19], the mean hematocrit was 32.3% in cats in IRIS stage 3 [16], and most of the other parameters were similar. The manifestations of feline CKD varied between individuals, thus there was a need for adjustment of therapy according to individual needs. The international consensus guidelines recommend managing hydration, diet, hypertension, anemia, proteinuria, and other treatments [1,2], which corelate exactly to the follow-up therapies in the present study. The characteristics on the baseline coexisting disorders in the present study were mostly consistent with the previous studies in the literature: (A) the prevalence of hyperthyroidism (9.7%) versus the prevalence in cats over 8 years of age in Germany (11.4%) [22] and in cats older than 9 years in Japan (8.9%) [23] and in the UK (6%) [24]; (B) the prevalence of diabetes mellitus (3.0%) versus the prevalence of 2.9% out of 561 cats in Spain [5]; (C) the prevalence of congestive heart failure (7.5%) versus the prevalence of hypertrophic cardiomyopathy (the most commonly diagnosed heart disease in cats, as comparative data for were not found congestive heart failure overall) in 14.6% of adult cats [25], 29.4% of 9-years-or-older senior cats [26], and 19.6% in another study [27]; (D) the prevalence of neoplasia (6.0%) versus the prevalence in cats with CKD of 4.2% [28]; and (E) the prevalence of pancreatitis (2.2%) was similar to 2.4% by following necropsy [29] (comparative data in cats with CKD were not found, most likely because pancreatitis is difficult to diagnose ante mortem). Taken together, all the baseline characteristics in this study suggested the population examined was very consistent with those in previously published studies in this field.

The findings on the progression-free survival analysis in the present study indicate that beraprost therapy will be associated with slower progression. The results of the no beraprost therapy group were very similar to those of a previous study on cats in IRIS stage 3 [15]. In the cohort A of this study, the progression-free survival rate at 1 year was 37.6%, disease progression was seen in 36 out of 77 cats (46.8%), death in 19 cats (24.7%) and euthanasia in 1 cat (1.3%); in the previous study, disease progression (defined as a 25% increase in creatinine concentration, the same as the present study) was seen in 34 out of 73 cats (46.6%) and death in 19 cats (26.0%). The progression-free survival rate and median progression survival time of the beraprost therapy group were 1.7 and 2.8 times, respectively, longer than those of the no beraprost therapy group, with a significant difference in the Kaplan–Meyer curves (*p* = 0.004 by log-rank test).

In addition, the data on the overall survival analysis demonstrated that beraprost was associated with prolonged life expectancy in cats with CKD, irrespective of coexisting disorders. The results of the no beraprost therapy group were within the range in the previously published literature, which tended to exclude the cats with coexisting disorders from the scope of research: (A) the overall survival rate at 3 years was 18.1% versus the survival rate of 12% at 2.6 years of cats with uremic CKD [19] and was 37% at 2.7 years in cats in IRIS stage 3 [16]; (B) the median overall survival time was 9.5 months versus the survival time of 7.8 months in cats with uremic CKD [19] and was 8.8 to 25.9 months in cats in IRIS stage 3 [16,30,31]. The overall survival rate and median overall survival time of the beraprost therapy group were 1.9 and 2.5 times, respectively, longer than those of the no beraprost therapy group, with a significant difference in the Kaplan–Meyer curves (*p* = 0.004 by log-rank test). Euthanasia is almost always regarded as a confounding factor in veterinary studies; cats are often euthanized rather than dying naturally [20], and 75% of cats were euthanized in one study on survival in cats with CKD [31]. In the present study, only 3 (2%) out of 134 cats were euthanized, which may have been due to geographical variation, but it introduced much less bias in the survival analysis.

Proteinuria in cats is often associated with CKD, with the prevalence reported to be 16% [30]. The severity of proteinuria at the time of diagnosis is also known to indicate a poorer prognosis [15,16,20,32,33]. Benazepril has been clinically tested in cats with CKD, shown to reduce proteinuria assessed by the urine-protein-to-creatinine ratio [34,35] and to decrease serum creatinine [36]; however, benazepril efficacy has not been demonstrated for reducing the incidences of death or withdrawal from the trial due to worsening CKD, reaching IRIS stage 4 [34], or the composite endpoint of death, euthanasia, or the need for parenteral fluid therapy [35]. Similarly, telmisartan has been shown to reduce the severity of proteinuria [37], but its efficacy has not been examined for slowing disease progression or prolonging survival. Collectively, the findings of the present study can be considered the first evidence for a medical therapy associated with an increased life expectancy in cats with CKD.

The onset profile of chronic disorders during the study period in the no beraprost therapy group was mostly consistent with the record of baseline coexisting disorders in the present study; however, there were some differences, in that neuromuscular disorders such as epilepsy and seizures were newly observed, and cardiovascular disorders increased during the study. Epilepsy and seizures in cats with CKD can be caused by uremic encephalopathy; it is reported that all cats with kidney encephalopathy had IRIS stage 3 or 4 CKD, with mean creatinine of 2.9 mg/dL [38]. Cardiovascular–renal disorders (CvRDs) are the pathophysiological relationships between kidneys and hearts in disease, and kidney injury is proposed to be able to lead to systemic volume overload that contributes to congestion, especially in animals with coexisting cardiac disease, including cardiomyopathy [3]; these factors could have influenced the results. Overall, the relative risk of onset of chronic disorders in the beraprost therapy group versus the no beraprost therapy group was 0.42 (8.8/20.8). Epilepsy and seizures were not seen in the beraprost therapy group but were seen in 3 cats (3.9%) in the no beraprost therapy group. Endothelial dysfunction and reduced NO production are associated with CKD and heart failure and make the link between kidney and cardiac dysfunction [39]. Beraprost has a protective effect on endothelial cell damage [9], and it especially, dose dependently inhibits human aortic endothelial cell injury induced by uremic toxin indoxyl sulfate [13] and stimulates eNOS in human and animal endothelial cells [10]. In the present study, the relative risk of incident cardiovascular disorders in the beraprost therapy group comparing the no beraprost therapy group was 0.34 (3.5/10.4). This finding may help to further elucidate the efficacy of beraprost in cats with CvRD. Additionally, there was no difference in the incidence of endocrine disorders and neoplasia between the groups.

While it is true that selection bias was minimized because all cats and their owners were proposed for a treatment of beraprost, and there was no special motivation, there may have been potential selection bias between the groups. The difference may have resulted from the difference in cats and their owners’ acceptability for the burdens and benefits (e.g., twice daily administration and costs).

In cohort A with IRIS stage 3 CKD (*n* = 134), the baseline phosphate was higher in the no beraprost therapy group, and subcohort B was sorted according to phosphate < 6.0 mg/dL (*n* = 97) to remove this difference. This allowed for balanced baseline phosphate between groups, but there remained differences in the level of urea, calcium, and the prescription of kidney diet-other (other than Renal support and k/d), the potential effects of which are unknown. It was also not possible to analyze a subcohort with phosphate ≥ 6.0 mg/dL due to the low sample size. These remain as important limitations of the study. Another limitation of the study is that the data used did not include enough information on blood pressure (the implementation rate of blood pressure measurements was 36.6%; most of cats did not undergo the routine assessment early in the study period) and hardly any on urine protein/creatinine ratio; thus, the prevalence of hypertension and proteinuria between the groups could not be accurately examined. In addition, it may be inappropriate to draw conclusions without considering the differences of treatment types on coexisting disorders. Also, the effects of beraprost on each coexisting disorder itself were not examined here. Endothelial dysfunction, a main target of beraprost, is known to be a key in the pathogenesis of various diseases such as heart disease and diabetes mellitus. Thus, it would be necessary to verify the current findings by a double-blinded, randomized, controlled clinical trial to address the above limitations.

In conclusion, beraprost therapy is associated with better overall survival in CKD feline patients in real-world practice.

## Figures and Tables

**Table 1:** Distribution of IRIS stage and beraprost prescription in the CKD-diagnosed cats.

			Total (*n* = 730)	Beraprost Prescription	
				Yes (*n* = 124)	No (*n* = 606)
IRIS	Stage 1	number	143	13	130
	Stage 2	number	369	37	332
	Stage 3	number	134	57	77
	Stage 4	number	84	17	67

**Table 2:** Baseline characteristics of all cats in cohort A: IRIS CKD stage 3 (creatinine: 2.9–5.0 mg/dL).

		Total (*n* = 134)	Beraprost Therapy (*n* = 57)	No Beraprost Therapy (*n* = 77)	*p* Value
Age (years)	median (25th, 75th percentile)	15.3 (10.5, 17.3)	15.9 (13.0, 17.3)	15.1 (8.6, 17.4)	0.254
Weight (kg)	median (25th, 75th percentile)	3.6 (3.0, 4.4)	3.8 (3.1, 4.4)	3.4 (2.8, 4.4)	0.142
Sex					
Female	number (%)	62 (46.3)	30 (52.6)	32 (41.6)	0.224
Male	number (%)	72 (53.7)	27 (47.4)	45 (58.4)	
Neutered	number (%)	115 (85.8)	46 (80.7)	69 (89.6)	0.210
Breeds					
Domestic Shorthair	number (%)	91 (67.9)	37 (64.9)	54 (70.1)	0.577
Japanese Bobtail	number (%)	7 (5.2)	3 (5.3)	4 (5.2)	
American Shorthair	number (%)	6 (4.5)	3 (5.3)	3 (3.9)	
Other	number (%)	30 (22.4)	14 (24.5)	16 (20.8)	
Biochemistry					
Creatinine (mg/dL)	median (25th, 75th percentile)	3.3 (3.0, 3.7)	3.3 (3.0, 3.7)	3.3 (3.0, 3.7)	0.767
Urea (mg/dL)	median (25th, 75th percentile)	42.0 (35.0, 60.0)	38.0 (31.0, 52.5)	46.0 (37.0, 70.0) **	0.001
Phosphate (mg/dL)	median (25th, 75th percentile)	4.9 (4.0, 6.1)	4.6 (3.9, 5.3)	5.3 (4.2, 6.8) **	0.006
Calcium (mg/dL)	median (25th, 75th percentile)	9.7 (9.2, 10.1)	9.8 (9.4, 10.4) *	9.6 (9.0, 10.0)	0.016
Potassium (mmol/L)	median (25th, 75th percentile)	3.9 (3.5, 4.2)	3.9 (3.6, 4.2)	3.9 (3.5, 4.3)	0.445
Hematology					
Packed cell volume (%)	median (25th, 75th percentile)	35.0 (30.0, 40.0)	36.0 (31.0, 41.0)	33.5 (29.5, 40.0)	0.075
Urinalysis					
Urine specific gravity	median (25th, 75th percentile)	1.014 (1.012, 1.018)	1.014 (1.012, 1.018)	1.014 (1.012, 1.018)	0.800
Urine protein (mg/dL)	mean (SD)	59.6 (167.9)	27.5 (60.1)	90.0 (223.6) *	0.048
Blood pressure measurement					
Systolic blood pressure (mmHg)	median (25th, 75th percentile)	153.0 (132.0, 165.0)	153.0 (132.0, 165.0)	151.5 (132.8, 160.3)	0.976
Treatment					
Beraprost (RAPROS)	number (%)	57 (42.5%)	57 (100.0%) **	0 (0.0%)	<0.001
Dose (μg/kg twice daily)	mean (SD, range)	15.0 (4.0, 7.1–26.2)	15.0 (4.0, 7.1–26.2) **	0 (0, 0–0)	<0.001
Subcutaneous fluid therapy (total)	number (%)	106 (79.1%)	42 (73.7%)	64 (83.1%)	0.203
At clinic	number (%)	87 (64.9%)	32 (56.1%)	55 (71.4%)	0.071
At home	number (%)	72 (53.7%)	28 (49.1%)	44 (57.1%)	0.385
Kidney Diet (total)	number (%)	75 (56.0%)	35 (61.4%)	40 (51.9%)	0.296
Royal Canin (Renal Support)	number (%)	53 (39.6%)	22 (38.6%)	31 (40.3%)	0.860
Hill’s (k/d)	number (%)	34 (25.4%)	16 (28.1%)	18 (23.4%)	0.553
Other	number (%)	33 (24.6%)	20 (35.1%) *	13 (16.9%)	0.025
Phosphate binder	number (%)	40 (29.9%)	16 (28.1%)	24 (31.2%)	0.849
Ferric chloride (Lenziaren)					
Oral activated charcoal	number (%)	37 (27.6%)	15 (26.3%)	22 (28.6%)	0.846
ACEI/ARB (total)	number (%)	34 (25.4%)	15 (26.3%)	19 (24.7%)	0.843
Benazepril (Fortekor)	number (%)	27 (20.1%)	10 (17.5%)	17 (22.1%)	0.664
Telmisartan (Semintra)	number (%)	10 (7.5%)	5 (8.8%)	5 (6.5%)	0.743
Calcium channel blocker	number (%)	11 (8.2%)	7 (12.3%)	4 (5.2%)	0.203
Amlodipine					
Erythrocyte-stimulating agents	number (%)	34 (25.4%)	11 (19.3%)	23 (29.9%)	0.228
Darbepoetin alfa					
Managing inappetence, nausea and vomiting (total)	number (%)	102 (76.1%)	41 (71.9%)	61 (79.2%)	0.413
Maropitant (Cerenia)	number (%)	88 (65.7%)	34 (59.6%)	54 (70.1%)	0.270
Mirtazapine	number (%)	44 (32.8%)	21 (36.8%)	23 (29.9%)	0.458
Famotidine	number (%)	27 (20.1%)	16 (28.1%)	11 (14.3%)	0.054
Omeprazole	number (%)	8 (6.0%)	2 (3.5%)	6 (7.8%)	0.466
Metoclopramide	number (%)	11 (8.2%)	5 (8.8%)	6 (7.8%)	1.000
Coexisting Disorders					
Any	number (%)	38 (28.4%)	18 (31.6%)	20 (26.0%)	0.562
Hyperthyroidism	number (%)	13 (9.7%)	7 (12.3%)	6 (7.8%)	0.395
Congestive heart failure	number (%)	10 (7.5%)	2 (3.5%)	8 (10.4%)	0.189
Neoplasia ^†^	number (%)	8 (6.0%)	4 (7.0%)	4 (5.2%)	0.723
Diabetes mellitus	number (%)	4 (3.0%)	3 (5.3%)	1 (1.3%)	0.312
Pancreatitis	number (%)	3 (2.2%)	2 (3.5%)	1 (1.3%)	0.575

Percentiles were identified by the Tukey’s hinges. Abbreviations: UPC, urine protein/creatinine ratio; ACEI, angiotensin converting enzyme inhibitor; ARB, angiotensin receptor blocker. Notes: RAPROS^®^, Toray Industries, Inc., Tokyo, Japan; Renal Support, Royal Canin Inc., Aimargues, France; k/d, Hill’s Pet Nutrition Inc., Topeka, KS, USA; Lenziaren and Fortekor, Elanco Animal Health Inc., Greenfield, IN, USA; Semintra, Boehringer Ingelheim Animal Health, Inc., Ingelheim, Germany; Cerenia, Zoetis Inc., Parsippany-Troy Hills, NJ, USA. ** *p* < 0.01 by Mann–Whitney U test for beraprost dose and Fisher’s exact test for beraprost, * *p* < 0.05, ** *p* < 0.01 by Mann–Whitney U test for urea, phosphate, calcium, urine protein, and beraprost dose, and Fisher’s exact test for beraprost. Percentiles were identified by the Tukey’s hinges. ^†^ Neoplasia in the beraprost therapy group included nasal lymphoma, kidney cell carcinoma, transitional cell carcinoma, and mammary cancer; neoplasia in the no beraprost therapy group included gastrointestinal lymphoma (in 3 cats) and liver cell carcinoma.

**Table 3:** Multivariable Cox regression analyses of progression-free and overall survival with baseline characteristics as covariates in cohort A: IRIS CKD stage 3 (creatinine: 2.9–5.0 mg/dL).

**Progression-Free Survival**						
**Covariate**	**Coefficient**	**Standard Error**	**Hazard** **Ratio for Disease Progression**	**95% Confidence Interval**		***p* Value**
				**Lower Limit**	**Upper Limit**	
Beraprost	−0.52	0.24	0.59	0.37	0.96	0.032 *
Urea (mg/dL) ≥ 60.0	1.06	0.27	2.90	1.72	4.89	<0.001 **
**Overall Survival**						
**Covariate**	**Coefficient**	**Standard Error**	**Hazard** **Ratio for Death**	**95% Confidence Interval**		***p* Value**
				**Lower Limit**	**Upper Limit**	
Beraprost	−0.54	0.27	0.58	0.34	0.99	0.047 *
Urea (mg/dL) ≥ 60.0	0.96	0.34	2.61	1.35	5.06	0.004 **
Phosphate (mg/dL) ≥ 7.0	0.88	0.46	2.41	0.98	5.94	0.056

* *p* < 0.05, ** *p* < 0.01 by Wald test.

**Table 4:** Subgroup analyses of progression-free and overall survival according to baseline in cohort A: IRIS CKD stage 3 (creatinine: 2.9–5.0 mg/dL).

**Progression-Free Survival**					
	**Beraprost Therapy versus** **No Beraprost Therapy**				
**Subgroups**	**Number of Cats**	**Hazard Ratio** **for Disease Progression**	**95% Confidence Interval**		***p* Value**
			**Lower Limit**	**Upper Limit**	
All	134	0.53	0.34	0.83	0.005 **
Age (years) ≤ 15.0	61	0.39	0.18	0.83	0.015 *
Weight (kg) ≥ 3.0	99	0.53	0.31	0.90	0.018 *
Creatinine (mg/dL) < 4.0	109	0.53	0.32	0.88	0.015 *
Urea (mg/dL) < 120.0	129	0.57	0.36	0.89	0.014 *
Phosphate (mg/dL) < 6.0	97	0.51	0.30	0.88	0.015 *
Calcium (mg/dL) < 10.6	116	0.48	0.30	0.78	0.003 **
Potassium (mmol/L) ≥ 3.5	104	0.59	0.35	0.98	0.042 *
Packed cell volume (%) ≥ 30.0	105	0.47	0.28	0.79	0.005 **
Urine protein (mg/dL) ≤ 300	112	0.60	0.37	0.97	0.037 *
**Overall Survival**					
	**Beraprost Therapy versus** **No Beraprost Therapy**				
**Subgroups**	**Number of Cats**	**Hazard Ratio** **for Death**	**95% Confidence Interval**		***p* Value**
			**Lower Limit**	**Upper Limit**	
All	134	0.49	0.30	0.81	0.005 **
Age (years) ≤ 15.0	61	0.25	0.10	0.67	0.006 **
Weight (kg) ≥ 3.0	99	0.45	0.24	0.81	0.008 **
Creatinine (mg/dL) < 4.0	109	0.45	0.25	0.80	0.007 **
Urea (mg/dL) < 120.0	129	0.53	0.32	0.88	0.014 *
Phosphate (mg/dL) < 6.0	97	0.46	0.25	0.86	0.015 *
Calcium (mg/dL) < 10.6	116	0.44	0.26	0.76	0.003 **
Potassium (mmol/L) ≥ 3.5	104	0.55	0.31	0.97	0.039 *
Packed cell volume (%) ≥ 30.0	105	0.42	0.23	0.75	0.004 **
Urine protein (mg/dL) ≤ 300	112	0.54	0.32	0.92	0.023 *

* *p* < 0.05, ** *p* < 0.01 by Wald test.

**Table 5:** Baseline characteristics of all cats in cohort B: IRIS CKD stage 3 (creatinine: 2.9–5.0 mg/dL) and phosphate < 6.0 mg/dL.

		Total (*n* = 97)	Beraprost Therapy (*n* = 47)	No Beraprost Therapy (*n* = 50)	*p* Value
Age (years)	median (25th, 75th percentile)	15.6 (12.0, 17.1)	15.9 (13.8, 17.1)	15.1 (9.5, 17.3)	0.302
Weight (kg)	median (25th, 75th percentile)	3.7 (3.0, 4.7)	3.9 (3.4, 4.7)	3.7 (2.9, 4.8)	0.337
Sex					
Female	number (%)	53 (54.6%)	27 (57.4%)	26 (52.0%)	0.684
Male	number (%)	44 (45.4%)	20 (42.6%)	24 (48.0%)	
Neutered	number (%)	85 (87.6%)	38 (80.9%)	47 (94.0%)	0.066
Breeds					
Domestic Shorthair	number (%)	66 (68.0%)	33 (70.2%)	33 (66.0%)	0.670
Japanese Bobtail	number (%)	5 (5.2%)	3 (6.4%)	2 (4.0%)	
American Shorthair	number (%)	6 (6.2%)	3 (6.4%)	3 (6.0%)	
Other	number (%)	20 (20.6%)	8 (17.0%)	12 (24.0%)	
Biochemistry					
Creatinine (mg/dL)	median (25th, 75th percentile)	3.2 (3.0, 3.5)	3.2 (3.0, 3.6)	3.2 (3.0, 3.4)	0.335
Urea (mg/dL)	median (25th, 75th percentile)	38.0 (32.0, 48.0)	36.0 (30.5, 44.0)	40.5 (35.3, 48.8) *	0.045
Phosphate (mg/dL)	median (25th, 75th percentile)	4.3 (3.8, 5.1)	4.3 (3.8, 5.0)	4.4 (3.9, 5.2)	0.280
Calcium (mg/dL)	median (25th, 75th percentile)	9.7 (9.2, 10.1)	9.8 (9.4, 10.4) *	9.5 (9.0, 10.0)	0.032
Potassium (mmol/L)	median (25th, 75th percentile)	3.9 (3.5, 4.2)	4.0 (3.7, 4.2)	3.9 (3.5, 4.2)	0.119
Hematology					
Packed cell volume (%)	median (25th, 75th percentile)	36.5 (32.0, 40.0)	37.0 (32.5, 41.0)	35.0 (31.0, 40.0)	0.146
Urinalysis					
Urine specific gravity	median (25th, 75th percentile)	1.014 (1.013, 1.017)	1.014 (1.013, 1.017)	1.014 (1.012, 1.018)	0.837
Urine protein (mg/dL)	mean (SD)	36.7 (116.6)	22.8 (51.5)	52.7 (161.3)	0.141
Blood pressure measurement					
Systolic blood pressure (mmHg)	median (25th, 75th percentile)	155.0 (139.0, 165.0)	158.0 (140.0, 166.0)	155.0 (137.5, 160.5)	0.456
Treatment					
Beraprost (RAPROS)	number (%)	47 (48.5%)	47 (100.0%) **	0 (0.0%)	<0.001
Dose (μg/kg twice daily)	mean (SD, range)	14.4 (3.6, 7.1–22.2)	14.4 (3.6, 7.1–22.2) **	0 (0, 0–0)	<0.001
Subcutaneous fluid therapy (total)	number (%)	76 (78.4%)	33 (70.2%)	43 (86.0%)	0.084
At clinic	number (%)	65 (67.0%)	27 (57.4%)	38 (76.0%)	0.083
At home	number (%)	48 (49.5%)	20 (42.6%)	28 (56.0%)	0.225
Kidney Diet (total)	number (%)	53 (54.6%)	28 (59.6%)	25 (50.0%)	0.416
Royal Canin (Renal Support)	number (%)	37 (38.1%)	17 (36.2%)	20 (40.0%)	0.835
Hill’s (k/d)	number (%)	24 (24.7%)	13 (27.7%)	11 (22.0%)	0.639
Other	number (%)	28 (28.9%)	19 (40.4%) *	9 (18.0%)	0.024
Phosphate binder	number (%)	24 (24.7%)	9 (19.1%)	13 (26.0%)	0.474
Ferric chloride (Lenziaren)					
Oral activated charcoal	number (%)	26 (26.8%)	13 (27.7%)	13 (26.0%)	1.000
ACEI/ARB (total)	number (%)	26 (26.8%)	12 (25.5%)	14 (28.0%)	0.822
Benazepril (Fortekor)	number (%)	19 (19.6%)	7 (14.9%)	12 (24.0%)	0.312
Telmisartan (Semintra)	number (%)	10 (10.3%)	5 (10.6%)	5 (10.0%)	1.000
Calcium channel blocker	number (%)	9 (9.3%)	7 (14.9%)	2 (4.0%)	0.085
Amlodipine					
Erythrocyte-stimulating agents	number (%)	21 (21.6%)	8 (17.0%)	13 (26.0%)	0.330
Darbepoetin alfa					
Managing inappetence, nausea and vomiting (total)	number (%)	77 (79.4%)	36 (76.6%)	41 (82.0%)	0.618
Maropitant (Cerenia)	number (%)	66 (68.0%)	29 (61.7%)	37 (74.0%)	0.276
Mirtazapine	number (%)	31 (32.0%)	18 (38.3%)	13 (26.0%)	0.276
Famotidine	number (%)	22 (22.7%)	14 (29.8%)	8 (16.0%)	0.146
Omeprazole	number (%)	6 (6.2%)	2 (4.3%)	4 (8.0%)	0.678
Metoclopramide	number (%)	10 (10.3%)	4 (8.5%)	6 (12.0%)	0.742
Coexisting Disorders					
Any	number (%)	24 (24.7%)	11 (23.4%)	13 (26.0%)	0.817
Hyperthyroidism	number (%)	10 (10.3%)	6 (12.8%)	4 (8.0%)	0.516
Congestive heart failure	number (%)	4 (4.1%)	0 (0.0%)	4 (8.0%)	0.118
Neoplasia ^†^	number (%)	6 (6.2%)	3 (6.4%)	3 (6.0%)	1.000
Diabetes mellitus	number (%)	2 (2.1%)	1 (2.1%)	1 (2.0%)	1.000
Pancreatitis	number (%)	2 (2.1%)	1 (2.1%)	1 (2.0%)	1.000

** *p* < 0.01 by Mann–Whitney U test for beraprost dose and Fisher’s exact test for beraprost, * *p* < 0.05 by Mann–Whitney U test for urea, Welch’s *t*-test for calcium, and Fisher’s exact test for kidney diet-other. Percentiles were identified by the Tukey’s hinges. ^†^ Neoplasia in the beraprost therapy group included nasal lymphoma, kidney cell carcinoma, and transitional cell carcinoma; neoplasia in the no beraprost therapy group included gastrointestinal lymphoma (in 3 cats).

**Table 6:** Onset of chronic disorders of the two groups in cohort A: IRIS CKD stage 3 (creatinine: 2.9–5.0 mg/dL).

		Total (*n* = 134)	Beraprost Therapy (*n* = 57)	No Beraprost Therapy (*n* = 77)
Any	number (%)	21 (15.7%)	5 (8.8%)	16 (20.8%)
Cardiovascular disorders (total)	number (%)	10 (7.5%)	2 (3.5%)	8 (10.4%)
Congestive heart failure	number (%)	4 (3.0%)	0 (0.0%)	4 (5.2%)
Cardiomyopathy	number (%)	6 (4.5%)	2 (3.5%)	4 (5.2%)
Endocrine disorders (total)	number (%)	2 (1.5%)	0 (0.0%)	2 (2.6%)
Hyperthyroidism	number (%)	1 (0.7%)	0 (0.0%)	1 (1.3%)
Diabetes mellitus	number (%)	1 (0.7%)	0 (0.0%)	1 (1.3%)
Neuromuscular disorders (total)	number (%)	3 (2.2%)	0 (0.0%)	3 (3.9%)
Epilepsy & Seizures	number (%)	3 (2.2%)	0 (0.0%)	3 (3.9%)
Neoplasia (total)	number (%)	6 (4.5%)	3 (5.3%)	3 (3.9%)
Mast cell tumor	number (%)	1 (0.7%)	1 (1.8%)	0 (0.0%)
Lymphoma	number (%)	1 (0.7%)	0 (0.0%)	1 (1.3%)
Squamous cell carcinoma	number (%)	1 (0.7%)	1 (1.8%)	0 (0.0%)
Fibrosarcoma	number (%)	1 (0.7%)	0 (0.0%)	1 (1.3%)
Pulmonary adenocarcinoma	number (%)	1 (0.7%)	1 (1.8%)	0 (0.0%)
Mesothelioma	number (%)	1 (0.7%)	0 (0.0%)	1 (1.3%)

## Data Availability

The raw data supporting the conclusions of this article will be made available by the authors, without undue reservation.
